# Utilizing digitized occurrence records of Midwestern feral *Cannabis sativa* to develop ecological niche models

**DOI:** 10.1002/ece3.11325

**Published:** 2024-07-11

**Authors:** Tori Ford, Ademola Aina, Shelby Ellison, Tyler Gordon, Zachary Stansell

**Affiliations:** ^1^ USDA‐Agricultural Research Service, Plant Genetic Resources Unit Geneva New York USA; ^2^ Department of Plant and Agroecosystem Sciences University of Wisconsin‐Madison Madison Wisconsin USA

**Keywords:** climate, ecological niche modeling, feral cannabis, germplasm

## Abstract

Hemp (*Cannabis sativa* L.) has historically played a vital role in agriculture across the globe. Feral and wild populations have served as genetic resources for breeding, conservation, and adaptation to changing environmental conditions. However, feral populations of Cannabis, specifically in the Midwestern United States, remain poorly understood. This study aims to characterize the abiotic tolerances of these populations, estimate suitable areas, identify regions at risk of abiotic suitability change, and highlight the utility of ecological niche models (ENMs) in germplasm conservation. The Maxent algorithm was used to construct a series of ENMs. Validation metrics and MOP (Mobility‐oriented Parity) analysis were used to assess extrapolation risk and model performance. We also projected the final projected under current and future climate scenarios (2021–2040 and 2061–2080) to assess how abiotic suitability changes with time. Climate change scenarios indicated an expansion of suitable habitat, with priority areas for germplasm collection in Indiana, Illinois, Kansas, Missouri, and Nebraska. This study demonstrates the application of ENMs for characterizing feral Cannabis populations and highlights their value in germplasm conservation and breeding efforts. Populations of feral *C. sativa* in the Midwest are of high interest, and future research should focus on utilizing tools to aid the collection of materials for the characterization of genetic diversity and adaptation to a changing climate.

## Graphical abstract

Data occurrences and ecological predictors were used to create an ecological niche model to characterize the abiotic tolerances of feral Midwestern *Cannabis sativa*. Suitable habitat for feral Cannabis in the Midwest is characterized by low‐lying, well‐drained, and disturbed soils near river beds, with more precipitation in early fall and extreme temperatures—indicative of adaptation to drought conditions. Areas at greatest risk to lose suitability are in the states of Kansas, Missouri, Illinois, Indiana, and South Dakota.

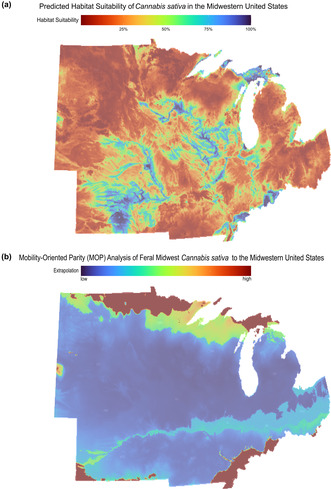

## 1 INTRODUCTION

Hemp, (*Cannabis sativa* L.) is among the oldest cultivated crops with documented usage dating to 6000 BCE, and has been widely and globally dispersed, broadly following human migration patterns (Clarke & Merlin, 2016; Jiang et al., 2016; Liu et al., 2017; McPartland & Small, 2020; Ren et al., 2021). Historic uses of the plant include fiber, grain, fuel, and secondary metabolites (Kobayashi et al., 2008; McPartland et al., 2019). During the United States' colonial period, industrial hemp was often produced via enslaved labor as a fiber commodity (Johnson, 2019), with large regions of the Southeast and Midwest dedicated to cordage and textile production. Policymakers and media entities linked “marihuana” with migrant labor communities and initiated policies culminating in 33 state‐wide bans by 1933, followed by effective full criminalization in the Marihuana Tax Act of 1937 (Johnson, 2019; Solomon, 2020). State and Federal political campaigns urging citizens to eradicate feral hemp populations were moderately successful; by 1938, approximately 26,000 tons of hemp were eradicated from 15,132 acres in 23 states, largely accomplished by civilians (Conrad, 1999; Johnson, 2019). Federal research continued despite these efforts; for example, communication between the Treasury Department, Narcotics Laboratory, and the University of Illinois Chemistry Department compared *Cannabis* with opiates and the Narcotics Laboratory provided large amounts of “wild” *Cannabis* for evaluation. During World War II, the U.S. Military faced significant long‐line fiber shortages and initiated the Federal “Hemp for Victory” Program (Filer, 2020; Johnson, 2019) to increase fiber supply. In 1942, American production was briefly revitalized from nearly zero to 226,000 acres by 1943 (Table S1; Hudson, 1942; Johnson, 2019). These efforts quickly diminished due to a lack of subsidies and federal regulation (Harmon, 2022; Johnson, 2019).

In the fall of the American hemp industry, remnants of the production lines remained as a growing population of feral escaped hemp gained recognition as “ditchweed.” Woods et al. (2023) determined that escaped American feral *C. sativa* are genetically distinct from other *C. sativa* populations. Populations collected from Nebraska, Kansas, and Colorado exhibit evidence of significant genetic variation indicative of local adaptation to abiotic and biotic factors (Woods et al., 2023), although evaluation by Carlson et al. (2021) indicated that some American feral populations most closely cluster with older European fiber landraces. Ren et al. (2021) also found that other American feral populations, from Kansas and Nebraska, closely cluster with landraces and feral germplasm from China.

Feral and wild populations of agronomic crops are typically valuable genetic resources to identify biotic and abiotic resistance, introgress unique alleles within crop breeding systems, and to address bottlenecks in genetic diversity (Henry & Nevo, 2014). These adapted populations could prove useful within breeding efforts for locally adapted cultivars. Plant breeding relies on collections of diverse germplasm to meet persistent challenges such as climate change, changing consumer demands, and evolving pathogen pressures (Byrne et al., 2018; Frankel, 1974; Mascher et al., 2019; Milner et al., 2019). For instance, East Asian rice (*Oryza sativa indica*) landraces held in germplasm repositories contributed dwarfing genes that allowed significant genetic gains during the Green Revolution (Hedden, 2003). Repositories also provided landrace wheat accessions with new sources of resistance (Gordon et al., 2021) to emerging races of a fungal pathogen of wheat, *Puccinia graminis* Pers.:Pers. f. sp. *tritici* Erikss. and E. Henning which had quickly evolved virulence on wheat cultivars with resistance genes that had been effective for decades (Singh et al., 2011). Conserving ex situ landraces, breeding lines, and feral accessions has the same potential in hemp. Maintaining diverse germplasm will allow for current and future breeding challenges to be addressed by the hemp breeding community. Current conservation efforts to collect, characterize, and conserve feral germplasm are ongoing (Ellison, 2021). These materials will be deposited within the newly formed Hemp Germplasm Collection administered by the United States Department of Agriculture National Plant Germplasm System which is now the largest repository of hemp genetic resources globally holding over 500 accessions, 128 of which are feral (USDA‐NPGS, Accessed Aug 8, 2023).

Ecological niche models (ENMs) estimate the dimensions of ecological niche space to predict geographic species distribution (Soberon & Peterson, 2005). ENMs have applications within paleoecology, distributional ecology, and conservation biology (Chiarenza et al., 2019; Martínez‐Freiría et al., 2020; Regos et al., 2021). ENMs generate predictive models by comparing species occurrence data against environmental predictors, where every point corresponds to a set of environmental suitability conditions (Hutchinson, 1957).

An ecological niche may be defined with various methods. Hutchinson delineates the niche into two categories: fundamental and realized niche, where the fundamental niche is described as the n‐dimensional hypervolume where every point corresponds to conditions deemed suitable for a species to persist, and the realized niche as the points of occurrence in physical space that match the fundamental niche (Hutchinson, 1957). Soberon and Peterson (2005) sought to alleviate the discrepancies in the definition and application of niches to ENMs with their biotic‐abiotic‐movement (BAM) diagram, defining the M region as the area accessible to the species since origin (Soberon & Peterson, 2005). ENMs are typically constructed to understand ecological niche on a species level; however, recent inquiries have been made questioning whether species level modeling was appropriate. Species level models tend to ignore locally adaptive response and advise that informing niche models using evolutionary relationships is key to truly understanding ecological niche space (Bothwell et al., 2021; Smith et al., 2019).

There are three main methodologies generating ENMs, presence–absence (PA), presence‐background (PB), and presence‐only (PO) and each relies on algorithms unique to the sampling methods. PA algorithms use presence–absence field samples to discriminate between environments of occupancy and nonoccupancy, providing probabilities of a species occurrence within an area. GAMs (generalized additive models), GLMs (generalized linear models), BRTs (boosted regression trees), and RFs (random forests) are PA models. PB algorithms only require presence occurrence data and that the study extent (accessible area) is large enough for a background sample to be representative of a species' niche; resulting models distinguish suitable and unsuitable habitat. Maxent (maximum entropy), GARP (genetic algorithm for rule‐set production), and ENFA (ecological niche factor analysis) are PB Models. PO requires only presence observations within an envelope algorithm, which defines potential niche but does not identify sinks properly, therefore making it less viable to identify areas of low suitability. Overlap Analysis, Bioclim, Domain, Habitat, and Mahalanobis Distance are PO models.

In practice, ENMs are mostly used for endangered species to make predictions of occurrence or persistence where the response to climate change remains unknown, and losing more germplasm would lead to critical endangerment (Borokini et al., 2023; Sony et al., 2018; Wani et al., 2021). However, this technology has been less explored in the context of diversifying crop germplasm for use in herbaria, ex situ conservation, and plant breeding.

Here, we develop an ecological niche model of a large subpopulation of Midwestern feral *C. sativa* to (i) characterize the abiotic conditions correlated with population occurrence, (ii) estimate the areas with the highest abiotic suitability for feral *C. sativa*, (iii) identify areas at the highest risk of abiotic suitability change in the short‐ (2021–2040) and mid‐term (2061–2080) future, (iv) highlight the utility of ENMs to germplasm collectors, and (v) utilize this technology to ultimately aid germplasm curators and plant breeders.

## 2 METHODS

### 2.1 Observance coordinates and data cleaning

Herbaria or occurrence coordinates were sourced from multiple online repositories (Table 1): Atlas of Living Australia (Belbin et al., 2021), Botanical Information and Ecology Network (Maitner, 2023), Centro de Referência em Informação Ambiental (Lima et al., 2021), Global Biodiversity Information Facility (Chamberlain et al., 2023), Genesys (Obreza, 2023), Germplasm Resources Information Network (USDA‐NPGS, accessed 02/2023), the Smithsonian National Museum of Natural History (2023, Smithsonian NMNH, accessed 01/2023), and vPlants (2001, accessed 04/2023). All online repositories were accessed through the APIs provided per source. Additional occurrence records were provided by collaborators at Cornell University, Kansas State University, and the University of Wisconsin Madison.

**TABLE 1:** Number of occurrences for each repository, before and after the cleaning process.

Repository	Precleaned occurrences	Final occurrences
University of Wisconsin‐ Madison	49	37
Botanical Information and Ecology Network (BIEN)	29	15
Cornell University	42	27
Global Biodiversity Information Facility (GBIF)	680	620
Germplasm Resources Information Network (GRIN‐Global)	86	53
Kansas State University	353	295
Smithsonian National Museum of Natural History	59	25
vPlants	590	121

**FIGURE 1:**
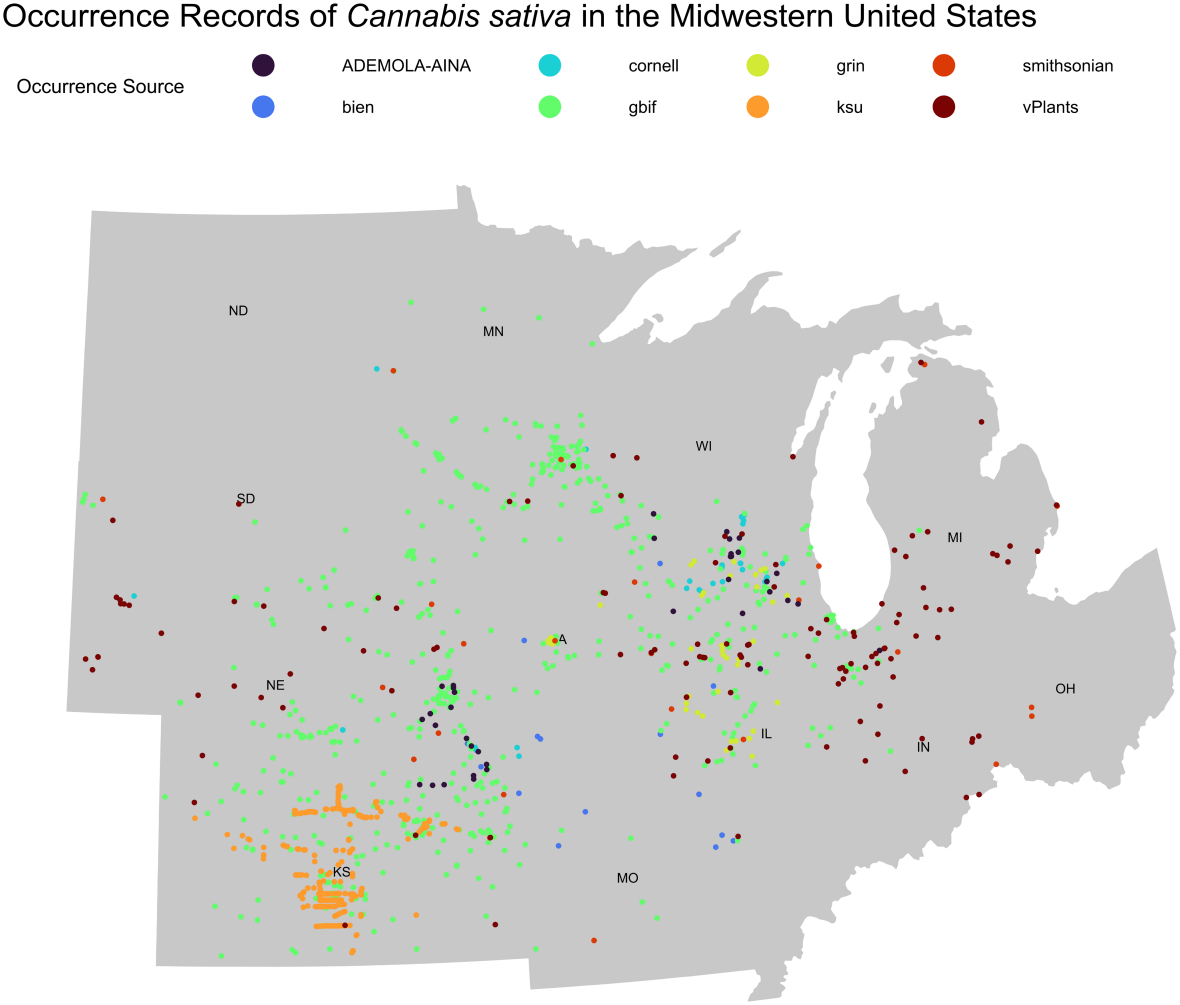
Map of *Cannabis sativa* occurrences in the Midwestern United States.

The occurrence records do not include absence data, so we selected a presence‐background algorithm such that the records defined the study extent across the Midwest. Occurrence records were cleaned to eliminate spatial biases by scrubbing duplicates, within and across repositories. Outliers, invalid coordinates, and records with institutional coordinates were removed. Occurrences were filtered to exclude any records not contained within the 12 Midwestern States (Figure 1, Table S2; Legind, 2018). Once restricted, occurrence records were thinned by 500 m (Sillero et al., 2021), resulting in 1193 cleaned, restricted, and thinned records (Table 1). Accessible area was limited to a 5 km buffered convex hull surrounding occurrences.

### 2.2 Ecological predictors

Ecological predictors represent the n‐dimensions in the Hutchinsonian fundamental niche and were sampled as rasters at varied resolutions. Ninety‐one variables from WorldClim (Fick & Hijmans, 2017), 18 Environmental Rasters for Ecological Modeling (ENVIREM, Title & Bemmels, 2018), and three variables from the U.S. Geological Survey Soil Properties dataset (Boiko et al., 2021) (Table S3) were downloaded. We calculated local daylength (the longest and shortest day) by latitude using the R package *geosphere* (Forsythe et al., 1995; Hijmans, 2022) as additional predictors to account for photoperiod sensitivity. In total, 115 environmental predictors were selected and clipped to the calibration and projection region.

### 2.3 Model development

Variance inflation factor (VIF) was used to detect multicollinearity within these ecological predictors. The *USDM*, “vifstep()” function was used to compute a VIF analysis for all predictors, reducing variables included in the final model to 19 (Table S4). All predictors with a VIF score over 10 were removed. AICc was selected as the optimal model‐selection method to explain the abiotic conditions suitable for this subpopulation, stronger transferability to project future climate scenarios, and overfitting resiliency (Warren et al., 2014). We also sought expert feedback on predictors selection from scientists collecting and evaluating these populations (A. Aina, personal communication, May 2023; Table S3). The Maxent algorithm was best to prioritize abiotic tolerance rather than the probability of occurrence. ENMs were constructed using the R package *ENMeval* (Kass et al., 2021) using the MaxEnt v3.4.4 (Phillips et al., 2006, 2017) algorithm. Occurrence records were used as presences and ecological predictors. Background points (*N* = 10,000) were sampled randomly within the study area as contingent absences resulting in a model that predicts potential niche (Lobo et al., 2010). Occurrence records and background records were spatially partitioned into equal quadrants called “blocks.” These blocks are spatially determined by the MaxEnt algorithm. The partitioned occurrence and background blocks serve as both testing and training datasets to generate average model validation statistics. Spatial partitions are recommended by Kass et al., specifically when processing large datasets, as random partitions can lead to artificially over‐performing models (2021). Block, and other spatial partitions like checkerboards, provide spatial cross‐validation which increases environmental extrapolation in the model that leads to models with higher transferability to new conditions; particularly useful when extrapolating to climate models (Kass et al., 2021). The Maxent algorithm returned models detailing the abiotic suitability of feral hemp across the study area. A total of 35 Maxent models were generated, with seven feature classes (L * Q * H) and five regularization (1:5) multipliers. Delta AICc scores were generated through ENMeval to compare the models.

The “ENMnulls()” function from *ENMeval* 2.0 (Kass et al., 2021) was used to compute 200 null model iterations. The validation statistics (AUC_test_, AUC_train_, AUC_diff_, OR_10p_, and CBI_val_) were visualized using evalplot.nulls() and values from the calibrated model were used for performance validation. The optimal model was projected to the Midwest using the *dismo* “predict()” function (Hijmans et al., 2022), and binary suitability maps were generated using MPA (Minimal Predicted Area) from the *ecospat* package (Di Cola et al., 2017). We set suitability thresholds for the percentage of included occurrences. Area suitability categories were defined by low to high ramping of the suitability threshold range. We generated response curves to describe the relationship of the environmental predictor to presence. MOP analysis was accomplished via the *kuenm* package (Cobos, Peterson, Barve, et al., 2019; Cobos, Peterson, Osorio‐Olvera, et al., 2019), “kuenm_mop()” by comparing environmental predictors between the calibration region and the Midwest United States.

### 2.4 Climate change scenarios

The optimal model was projected into the GISS‐E2‐1‐H climate scenario during two time periods, under two SSPs (Shared Socio‐Economic Pathways). SSPs represent future climate scenarios as a result of the intersection of political climate and social change (Hausfather, 2018). We projected the best model against two climate‐change scenarios using the *dismo's “*predict()” function and GISS‐E2‐1‐H climate model against SSP1 (+2.6°C; “Sustainability—Taking the Green Road”), SSP5 (+8.5°C; “Fossil‐fueled Development—Taking the Highway”) (Riahi et al., 2017) and we ran these models under two time periods: 2021–2040 and 2061–2080. The five models (current, normal, negligible, terrible, and meltdown) were converted to binary and compared with *ecospat's* “BIOMOD_RangeSize()” function, and the resulting comparisons were used to visualize trends in abiotic suitability across climate‐change scenarios.

## 3 RESULTS

### 3.1 Model validation

Of 35 models output by Maxent, the optimal model selected has an LH feature class with a regularization multiplier of 1. Accordingly, the AUC_train_ of the null models is significant (Figure S1). The AUC of the optimal model is 0.73, indicating good classification capacity. The CBI of the model is 0.64, indicating high model calibration and the null models indicate that CBI is significant. OR_10p_ did not meet the 0.05 significance threshold while AUC_diff_ surpassed the threshold (Figure S1). The larger pool of AUC_diff_ scores ranged from 0.031 to 0.098 and the optimal model had an AUC_diff_ of 0.089, which does not indicate severe overfitting (Bohl et al., 2019). We confidently state that the optimal model has good discriminatory abilities with some potential of being overfit.

### 3.2 Projection

**FIGURE 2:**
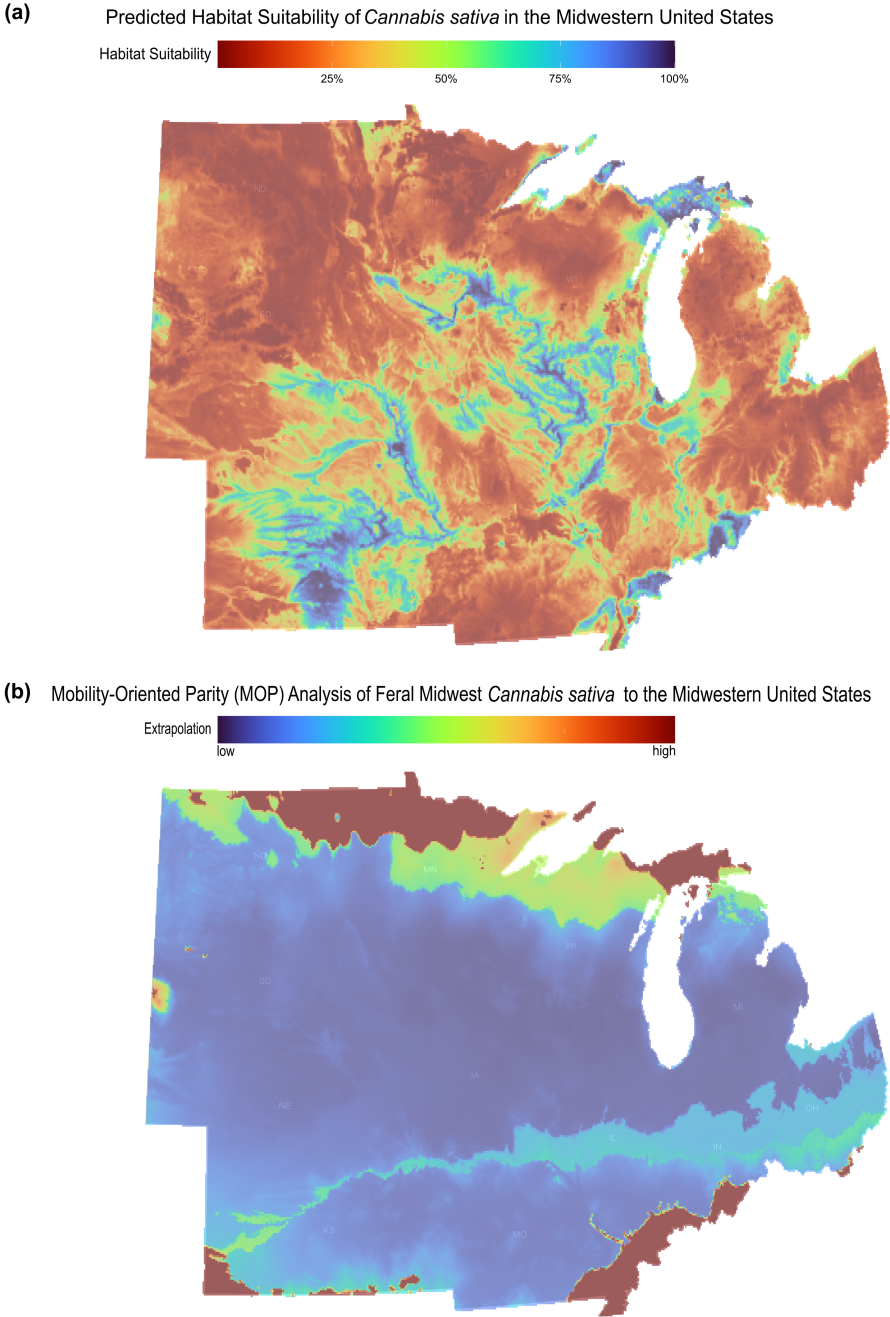
(a) Abiotic suitability heatmap for *Cannabis sativa* in the Midwestern United States. Areas in blue represent high abiotic suitability, and areas in red represent low abiotic suitability. (b) Mobility‐oriented Parity Analysis of the Calibration Region to the Entire Midwestern United States. Areas in blue and green represent low extrapolation, and areas in orange and red represent high extrapolation.

When projecting the model across the Midwest, the highest suitability areas lie along low‐lying river channels and tributaries, specifically the Upper Mississippi, Ohio, Missouri, and Platte Rivers. In fact, the only suitable area at the highest suitability threshold lies along river channels and the coasts of the Great Lakes (Figure 2a). The largest continuous region of abiotic unsuitability was located within northern Minnesota, predominately within Aitkin, Beltrami, Koochiching, and Itasco counties. Another large region of abiotic unsuitability was located within North Dakota (Haakon, Jones, Lyman, and Stanley counties). MOP analysis indicated the areas under the highest risk of unreliable prediction (extrapolation) are the Northern half of North Dakota, Minnesota, Wisconsin, and Michigan's Upper Peninsula and Ohio River regions (southern Missouri, Illinois, and Indiana) (Figure 2b).

The optimal habitat of feral *C. sativa* occurrence is defined by the most important ecological predictors of occurrence determined by the model. The predictor most effective for predicting the occurrence of feral hemp was BIO8 (mean temperature of the driest quarter); the driest quarter is the winter months, December, January, and February (MRCC, 2024). Response curves indicate that feral hemp occurrence is higher in areas characterized by temperature extremes during these months. The second‐best ecological predictor of occurrence was fc_gNATSGO_US (soil field capacity). Response curves indicate that lower field capacity (e.g., the water content retained after excess water drainage) predicted feral hemp occurrence. The third‐best ecological predictor of occurrence was PREC09 (average precipitation in September). Response curves indicate that high precipitation in September predicted feral hemp occurrence. In the Midwest, September is the last month of the year averaging more than 1 day of moderate rainfall (over 0.5 inches; MRCC, 2024). As drought conditions persist in the Midwest (National Oceanic and Atmospheric Administration, 2023), September's precipitation is crucial for providing season‐extending irrigation for nonirrigated or drought‐stricken plant populations (Goldstein, 2023).

### 3.3 Climate change predictions

**FIGURE 3:**
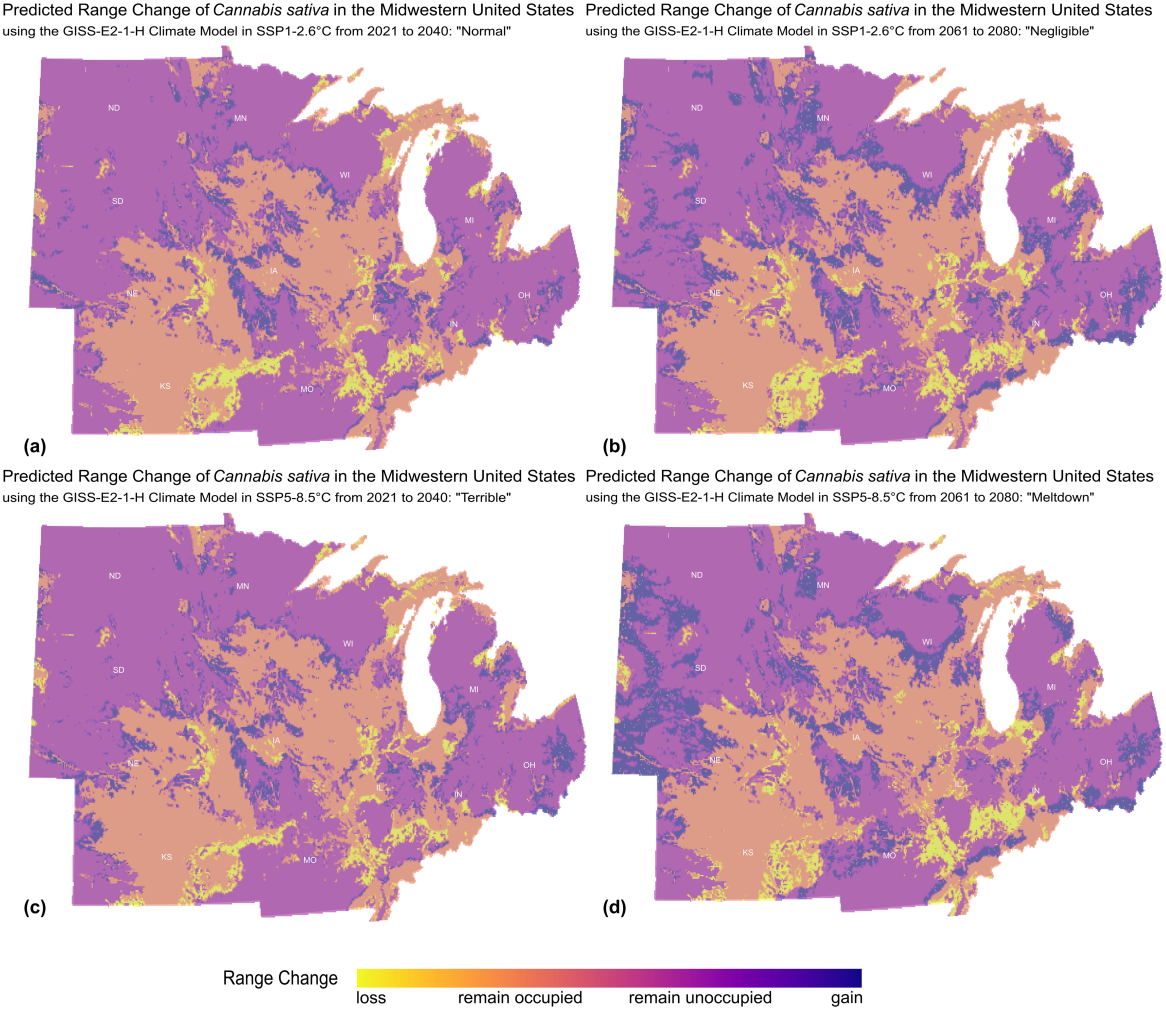
Range comparisons of loss, occupation, inoccupation, and gain under the four climate change scenarios—normal (a), negligible (b), terrible (c), and meltdown (d)—compared to the current conditions.

Generally, climate change scenario models suggest an increase in total abiotic suitable habitat for *Cannabis* and an expansion of that suitable area over time. Across all scenarios, primary states for habitat loss, and priority for germplasm collection, are Indiana, Illinois, Kansas, Missouri, and Nebraska. Generally, this means that these areas will observe a change in the ecological predictors associated with the persistence of feral hemp populations. Gains in suitability vary per climate scenario (Figure 3). For SSP1‐2.6°C, abiotic suitability was mainly gained in Iowa and temporarily in Ohio, with suitability drastically increasing into the far future. For SSP5‐8.5°C, the average predicted gain across the Midwest is 34% suitability from 2021 to 2080. The SSP5‐8.5°C when compared with SSP1‐2.6°C resulted in a greater increase in suitable land area. SSP5 gains more suitability than SSP1 in short‐ to mid‐term temporal projections (Table 3).

## 4 DISCUSSION

### 4.1 Study population

Hemp landraces have been poorly characterized and maintained which has likely led to the loss of genetic diversity. Admixture of feral, grain, fiber, and secondary metabolite cultivars may exacerbate genetic erosion (Torkamaneh & Jones, 2021). We have created an ecological niche model that describes the abiotic tolerances of an important subpopulation of *C. sativa*. We focused on the United States. Midwest feral hemp subpopulation for several reasons: significant population stratification is present in *C. sativa* and artificial selective pressure is considered to be high within distinct pools of *C. sativa* germplasm (e.g., temperate or equatorial populations have likely adapted to divergent abiotic conditions). To our knowledge—through queries on Google Scholar and Web of Science—there have been no previous attempts to create an ecological niche model of feral *C. sativa*, and these efforts can be used to inform ongoing feral collection efforts and enhance the conservation of hemp genetic resources. This currently understudied population of Midwestern *C. sativa* should be a top priority for conservation efforts.

### 4.2 Diversifying the approach

Although Maxent has been widely utilized within the ecological niche model space, computing these models under multiple other algorithms is a best practice. Follow‐up field sampling of these suitability predictions could be used as inputs within a subsequent presence–absence approach. Using predictive models to inform collections is an underutilized practice (Amici et al., 2014) and feral plants are an important group to study given that dispersal is partially driven through anthropological means.

### 4.3 Model results and implications

The current projection of the potential niche depicts high abiotic suitability mainly along river channels. The most influential environmental predictors concur with previous inquiries into the environmental conditions most suitable for the “naturalization” of hemp in Iowa. Haney and Kutscheid (1975) remark that “naturalized,” or feral hemp grows larger when in soils that are well‐drained with ample moisture. They also note that site disturbance was a predictor of feral hemp, observing populations along waste areas like fence rows, stream banks, ditches, and abandoned fields (Haney & Kutscheid, 1975), which likely corresponds with favored areas often corresponding with higher human population density.

*Cannabis sativa* cultivation in the Midwest typically entails a late May or early June transplantation. Young hemp plants are prone to low establishment if moisture and weed‐pest pressure are high. For a successful transplantation, *C. sativa* plants should be put in warm, well‐drained mostly loamy soils (Phillip Alberti, 2021). A review by Clarke and Merlin (2015) outlines the physiological needs of feral or “ruderal” hemp. They remark that wild‐type hemp generally is more tolerant to a wider range of climates than its domestic counterparts. They propose that “ruderal” plants rely on vastly deep root architecture and loose‐textured soils to withstand drought conditions and persist without the inputs of conventional agriculture. From our model, we observe the high contribution of soil field capacity on the model as a predictor (Table 2). Our model reports that the lower the field capacity, meaning soils retain less water, the higher the predicted occurrence of feral hemp. The model does not state that low field capacity is causative of feral hemp occurrence, it is correlated with higher occurrence.

**TABLE 2:** Top 10 environmental predictors contributing to the optimal model, in order of highest contribution to lowest.

Variable name	Contribution (%)	Permutation importance
Mean temperature of wettest quarter (BIO8)	41.26	8.83
Soil field capacity (fc_gNATSGO)	23.13	20.76
Average precipitation in month 09 (prec_09)	7.50	4.44
Average precipitation in month 01 (prec_01)	4.90	19.73
Sum of months with a mean temperature over 5°C × Number of days (growingDegDays5)	4.85	1.91
Elevation	3.67	16.91
Average solar radiation in month 04 (prec_04)	3.56	1.96
Mean monthly potential evapotranspiration of the driest quarter (PETDriestQuarter)	2.21	2.68
Terrain roughness index (tri)	2.15	3.55
Soil available water capacity (awc_gNATSGO)	1.74	2.83

*Note*: Percent contribution is the increase in regularized gain, per every iteration of developing the optimal model, converted to percentage. Permutation importance measures the decrease in AUC_train_ by permuting values of the training points in each variable after the model has been generated and converted to percentage (Phillips, 2017).

### 4.4 Models and their limitations

Models are not and should not be the end goal but are a useful tool to inform conservation and policy decisions. Our model evaluation statistics indicate the optimal model sufficiently predicts areas of abiotic suitability for *C. sativa* across the Midwest, however the limitations of this model must be further evaluated. Ecological predictors, WorldClim's bioclimatic variables, are well documented for being highly multicollinear (Arif et al., 2007). Some studies address this issue by removing multicollinear variables (Cobos, Peterson, Barve, et al., 2019; Cobos, Peterson, Osorio‐Olvera, et al., 2019) entirely while other studies suggest using a priori‐determined selection method (Zeng et al., 2016). A purely algorithmic‐based selection could select variables that result in a predictively accurate model with variables that are realistically uninfluential (Smith & Santos, 2020). A priori selection could result in variables that are highly multicollinear, resulting in an ultimately ineffective model (Cobos, Peterson, Barve, et al., 2019; Cobos, Peterson, Osorio‐Olvera, et al., 2019). We followed alternative recommendations by Smith and Santos (2020) to first select variables a priori, followed by algorithmic selection to remove multicollinear variables. By employing the VIF (variance inflation factor) analysis for algorithmic selection, we identified the variance induced when an ecological predictor is manipulated and allowed for comparisons of predictor collinearity (Cobos, Peterson, Barve, et al., 2019; Cobos, Peterson, Osorio‐Olvera, et al., 2019). Generally, VIF >10 is a signal of a model's excessive multicollinearity problem (Naimi et al., 2014).

From the MOP analysis, we also know that the optimal model does have highly extrapolative regions. Extrapolation in the model specifically refers to the model's capability to provide reliable predictions in areas that are too different from the areas in which the model is trained. This indicates that there is a gap in knowledge of the ability of feral hemp to grow in these areas, increasing sampling in these areas would lead to better predictive models.

Also, we did not include any anthropogenic factors in this study. Even though suitability expands with more extreme climate change scenarios, anthropogenic population shifts will likely occur during these times as well. Given that the dispersal of new feral populations is largely anthropologically driven, our models are not able to account for dispersal events by humans. Also, with few genotypes available within these populations, there is no way to distinguish what populations are remnants of historical hemp cultivation in the Americas, or European fiber, illicit high‐THC types, etc. For example, we did not evaluate the extent of the correlation between predicted biologically suitable habitat and historical hemp production regions. We also are not able to account for biotic interactions within the ENM, how those biotic factors would respond to climate change, and how those interactions shift in reaction to climate change.

### 4.5 Future models and their implications

Under short‐ and long‐term predictions of low and severe climate scenarios, there is always an expansion in the potential niche for feral hemp. We observe range change in two ways: by examining range size in the future with and without dispersal. Dispersal refers specifically to the expansion of current feral populations and new anthropologically driven feralization events. Range size with dispersal allows us to describe the potential niche of feral *Cannabis* under every scenario (Table 3). At full dispersal, the potential niche of feral *Cannabis* expands under every scenario but expands most during the “meltdown” climate change scenario, SSP5‐8.5°C from 2061 to 2080 (Figure 3d). Based on predictions of the potential expansion in abiotic suitability, this population could allow us to observe the contested Niche Variation Hypothesis, where populations with wider niches tend toward greater genetic variation (Van Valen, 1965). We can begin to ask questions such as: in areas where models predict niche expansion, do we observe more genotypic variation? The niche variation hypothesis asserts that there will be. As we continue to generate models, we should consider how niche variation is informed by genetics and use that lens to develop more complex models and hypotheses. As current research is underway to genotype and characterize the Midwestern feral *C. sativa* type (Ellison, 2021), these efforts will allow for more utilitarian predictive models.

**TABLE 3:** Pixel‐to‐pixel comparison of each climate scenario to the current predicted suitable area in the Midwest.

Climate model	Loss (pixels)	Remain occupied (pixels)	Remain unoccupied (pixels)	Gain (pixels)	% Loss	% Gain	% Species range change	Current range size (pixels)	Future range size with no dispersal	Future range size with full dispersal
GISS‐E2‐1‐H 2021‐2040 SSP1‐2.6C	6286	71,051	39,878	10,173	13.62	22.04	+8.42	46,164	39,878	50,051
GISS‐E2‐1‐H 2061‐2080 SSP1‐2.6C	6950	63,386	39,214	17,838	15.06	38.64	+23.59	46,164	39,214	57,052
GISS‐E2‐1‐H 2021‐2040 SSP5‐8.5C	5721	70,402	40,443	10,822	12.39	23.44	+11.05	46,164	40,443	51,265
GISS‐E2‐1‐H 2061‐2080 SSP5‐8.5C	6992	60,874	39,172	20,350	15.15	44.08	+28.94	46,164	39,172	59,522

The regions of highest priority for germplasm collection are the regions that experience suitability loss under both the “normal” and “terrible” climate change scenarios. The main areas of concern for feral Cannabis population loss over the next 20 years are in (i) Kansas—in the Flint Hills, South of Topeka and East of Wichita, (ii) Illinois and Missouri—along the Mississippi River, around Chester and St. Louis, respectively, (iii) Illinois—along the Fairfield basin and the cities of Naperville, Aurora, Rockford, Rochelle, LaSalle, and Rock Falls, (iv) Indiana—along the East Fork White River, and (v) the city of Bloomington, South Dakota—surrounding the Black Hills. Collections of these populations are critical to saving this germplasm in the case of a climate “meltdown”. Lower priority for germplasm collection regions is within Michigan, Minnesota, Ohio, and Iowa.

Future hemp breeding efforts will rely on diverse genetic resources conserved in seed banks to achieve genetic gain and address important agricultural challenges including climate change and emerging pest and disease pressures. Milner et al. (2019) demonstrated that affordable high‐throughput genotyping provided defining characteristics of an accession and population structure analyses differentiated accessions according to domestication status and geographic origin within the German barley collection. This information can define subpopulation pools for breeding, and combined with extensive phenotyping of accessions, can be used to identify potential duplicate accessions and germplasm gaps within collections. Therefore, genotyping accessions as they become available will be an early priority for newly acquired feral accessions identified in habitats through this modeling work.

Typically, seedbank accessions include passport records with geographic origin information and information describing the taxonomy and breeding history of each accession. However, standardized phenotyping protocols, like the newly developed USDA Hemp Descriptor and Phenotyping Handbook (https://www.ars.usda.gov/northeast‐area/geneva‐ny/plant‐genetic‐resources‐unit‐pgru/docs/hemp‐descriptors/), will also be essential tools for accession characterization. Phenotyping will be required for new germplasm acquisitions to identify highly heritable phenotypic traits that will be the focus of future breeding objectives. With the ongoing characterization of feral Cannabis, the germplasm collected could also aid in the development of standardization of phenotyping methods and sequencing protocols. In creating standards, breeders could accelerate their breeding programs.

## 5 CONCLUSIONS AND QUESTIONS

Due to the unique history of *Cannabis sativa* and its interaction with humans, we underscore the importance of this subpopulation as a useful case study to illuminate anthropogenic, genetic, environmental, and climate‐change interactions. We believe that the tools implemented in this work are currently underutilized by germplasm collectors, curators, and breeders and have applications within ongoing collection, conservation, characterization, and breeding efforts. Specifically, these ENMs or other predictive models could be critical to informing feral germplasm collection strategies, protocols, and priorities. Delineation of current and future species niche can inform cultural production practices and breeding objectives for enhanced abiotic tolerance.

## AUTHOR CONTRIBUTIONS

**Tori Ford:** Conceptualization (lead); methodology (lead); validation (lead); visualization (lead); writing – original draft (equal); writing – review and editing (equal). **Ademola Aina:** Data curation (supporting); writing – review and editing (supporting). **Shelby Ellison:** Writing – review and editing (supporting). **Tyler Gordon:** Supervision (equal); writing – review and editing (lead). **Zachary Stansell:** Conceptualization (lead); supervision (equal); writing – review and editing (lead).

## Supporting information


Data S1

## Data Availability

The data that support the findings of this study are openly available on GitHub at [https://github.com/tr‐ford/USDA_Cannabis_sativa_Midwest_Feral_Biogeo.git], reference: 25, Ford and Stansell (2023).
