# Supplementary material for: Utilizing digitized occurrence records of Midwestern feral Cannabis sativa to develop ecological niche models
Source: Ecol Evol. 2024 Jul 11;14(7):e11325. doi: 10.1002/ece3.11325 (PMC11239322; doi:10.1002/ece3.11325)
Supplement: Supplementary file 1 — Data S1 [file ECE3-14-e11325-s001.docx]

**Supplemental Information for:**

**Biogeography Project**

Tori Ford, Zachary Stansell, Shelby Ellison, Ademola Aina, Tyler Gordon

**Table of Contents:**

| **Hemp Production Comparison: Table 1** | Page 1 - 2 |
| --- | --- |
| **Occurrences by State: Table 2** | Page 2 |
| **Environmental Predictors: Table 3** | Page 2 - 4 |
| **VIF Selected Ecological Predictors: Table 4** | Page 4 - 5 |
| **Null Model Results: Figure 1** | Page 6 |
| **Top 4 Variable Importance Response Curves: Figure 2** | Page 7 |
| **PCA of the Top 10 Variables: Figure 5** | Page 8 |
| **Reasoning for Model Evaluation: Reasoning 1** | Page 8 - 9 |

Table 1.

Hemp Acreage compared between 1942 and 1943 by States Enrolled in the Hemp for Victory Program, derived from correspondence between the United States Department of Agriculture’s Commodity Credit Corporation and the secretary of War Hemp Industries, Inc. (WHI).

| Hemp for Victory Enrolled State | Acreage in 1942 | Acreage in 1943 |
| --- | --- | --- |
| Illinois | 500 | 60,000 |
| Indiana | 0 | 32,000 |
| Iowa | 0 | 60,000 |
| Kentucky | 5,000 | 48,000 |
| Minnesota | 500 | 60,000 |
| Wisconsin | 7,000 | 40,000 |

Table 2. Number of Feral *Cannabis sativa* Occurrence Records in Each Midwestern State

| State | Occurrences |
| --- | --- |
| Missouri | 97 |
| Iowa | 177 |
| Illinois | 169 |
| Wisconsin | 147 |
| Nebraska | 188 |
| North Dakota | 61 |
| Indiana | 98 |
| Michigan | 77 |
| South Dakota | 92 |
| Minnesota | 204 |
| Kansas | 518 |
| Ohio | 61 |

Table 3. All Ecological Predictors Considered for Use within the ENM

| **Database** | **Environmental Predictor** | **Description** | **References (Authors Name, Year)** |
| --- | --- | --- | --- |
| **WorldClim v2.1** | Elev | Elevation derived from the Shuttle Radar Topography Mission (SRTM) | *Fick and Hijmans,* [*2017*](https://worldclim.org/data/worldclim21.html) |
| **WorldClim v2.1** | BIO1 | Annual Mean Temperature | *Fick and Hijmans,* [*2017*](https://worldclim.org/data/worldclim21.html) |
| **WorldClim v2.1** | BIO2 | Mean Diurnal Range (Mean of monthly (max temp - min temp)) | *Fick and Hijmans,* [*2017*](https://worldclim.org/data/worldclim21.html) |
| **WorldClim v2.1** | BIO3 | Isothermality (BIO2/BIO7) (×100) | *Fick and Hijmans,* [*2017*](https://worldclim.org/data/worldclim21.html) |
| **WorldClim v2.1** | BIO4 | Temperature Seasonality (standard deviation ×100) | *Fick and Hijmans,* [*2017*](https://worldclim.org/data/worldclim21.html) |
| **WorldClim v2.1** | BIO5 | Max Temperature of Warmest Month | *Fick and Hijmans,* [*2017*](https://worldclim.org/data/worldclim21.html) |
| **WorldClim v2.1** | BIO6 | Min Temperature of Coldest Month | *Fick and Hijmans,* [*2017*](https://worldclim.org/data/worldclim21.html) |
| **WorldClim v2.1** | BIO7 | Temperature Annual Range (BIO5-BIO6) | *Fick and Hijmans,* [*2017*](https://worldclim.org/data/worldclim21.html) |
| **WorldClim v2.1** | BIO8 | Mean Temperature of Wettest Quarter | *Fick and Hijmans,* [*2017*](https://worldclim.org/data/worldclim21.html) |
| **WorldClim v2.1** | BIO9 | Mean Temperature of Driest Quarter | *Fick and Hijmans,* [*2017*](https://worldclim.org/data/worldclim21.html) |
| **WorldClim v2.1** | BIO10 | Mean Temperature of Warmest Quarter | *Fick and Hijmans,* [*2017*](https://worldclim.org/data/worldclim21.html) |
| **WorldClim v2.1** | BIO11 | Mean Temperature of Coldest Quarter | *Fick and Hijmans,* [*2017*](https://worldclim.org/data/worldclim21.html) |
| **WorldClim v2.1** | BIO12 | Annual Precipitation | *Fick and Hijmans,* [*2017*](https://worldclim.org/data/worldclim21.html) |
| **WorldClim v2.1** | BIO13 | Precipitation of Wettest Month | *Fick and Hijmans,* [*2017*](https://worldclim.org/data/worldclim21.html) |
| **WorldClim v2.1** | BIO14 | Precipitation of Driest Month | *Fick and Hijmans,* [*2017*](https://worldclim.org/data/worldclim21.html) |
| **WorldClim v2.1** | BIO15 | Precipitation Seasonality (Coefficient of Variation) | *Fick and Hijmans,* [*2017*](https://worldclim.org/data/worldclim21.html) |
| **WorldClim v2.1** | BIO16 | Precipitation of Wettest Quarter | *Fick and Hijmans,* [*2017*](https://worldclim.org/data/worldclim21.html) |
| **WorldClim v2.1** | BIO17 | Precipitation of Driest Quarter | *Fick and Hijmans,* [*2017*](https://worldclim.org/data/worldclim21.html) |
| **WorldClim v2.1** | BIO18 | Precipitation of Warmest Quarter | *Fick and Hijmans,* [*2017*](https://worldclim.org/data/worldclim21.html) |
| **WorldClim v2.1** | BIO19 | Precipitation of Coldest Quarter | *Fick and Hijmans,* [*2017*](https://worldclim.org/data/worldclim21.html) |
| **WorldClim v2.1** | tmin_1-12 | Temperature minimums, Jan-Dec | *Fick and Hijmans,* [*2017*](https://worldclim.org/data/worldclim21.html) |
| **WorldClim v2.1** | tmax_1-12 | Temperature maximums, Jan-Dec | *Fick and Hijmans,* [*2017*](https://worldclim.org/data/worldclim21.html) |
| **WorldClim v2.1** | prec_1-12 | Precipitation from Jan-Dec | *Fick and Hijmans,* [*2017*](https://worldclim.org/data/worldclim21.html) |
| **WorldClim v2.1** | srad_1-12 | Solar radiation from Jan-Dec | *Fick and Hijmans,* [*2017*](https://worldclim.org/data/worldclim21.html) |
| **WorldClim v2.1** | wind_1-12 | Wind speeds from Jan-Dec | *Fick and Hijmans,* [*2017*](https://worldclim.org/data/worldclim21.html) |
| **WorldClim v2.1** | vapr_1-12 | Water vapour pressure from Jan-Dec | *Fick and Hijmans,* [*2017*](https://worldclim.org/data/worldclim21.html) |
| **ENVIREM** | annualPET | annual potential evapotranspiration: a measure of the ability of the atmosphere to remove water through evapotranspiration processes, given unlimited moisture | *Title and Bemmels,* [*2018*](http://envirem.github.io/) |
| **ENVIREM** | aridityIndexThornthwaite | Thornthwaite aridity index: Index of the degree of water deficit below water need | *Title and Bemmels,* [*2018*](http://envirem.github.io/) |
| **ENVIREM** | climaticMoistureIndex | a metric of relative wetness and aridity | *Title and Bemmels,* [*2018*](http://envirem.github.io/) |
| **ENVIREM** | continentality | average temp. of warmest month - average temp. of coldest month | *Title and Bemmels,* [*2018*](http://envirem.github.io/) |
| **ENVIREM** | embergerQ | Emberger's pluviothermic quotient: a metric that was designed to differentiate among Mediterranean type climates | *Title and Bemmels,* [*2018*](http://envirem.github.io/) |
| **ENVIREM** | growingDegDays0 | sum of mean monthly temperature for months with mean temperature greater than 0℃ multiplied by number of days | *Title and Bemmels,* [*2018*](http://envirem.github.io/) |
| **ENVIREM** | growingDegDays5 | sum of mean monthly temperature for months with mean temperature greater than 5℃ multiplied by number of days | *Title and Bemmels,* [*2018*](http://envirem.github.io/) |
| **ENVIREM** | maxTempColdestMonth | max. temp. of the coldest month | *Title and Bemmels,* [*2018*](http://envirem.github.io/) |
| **ENVIREM** | minTempWarmestMonth | min. temp. of the warmest month | *Title and Bemmels,* [*2018*](http://envirem.github.io/) |
| **ENVIREM** | monthCountByTemp10 | count of the number of months with mean temp greater than 10℃ | *Title and Bemmels,* [*2018*](http://envirem.github.io/) |
| **ENVIREM** | PETColdestQuarter | mean monthly PET of coldest quarter | *Title and Bemmels,* [*2018*](http://envirem.github.io/) |
| **ENVIREM** | PETDriestQuarter | mean monthly PET of driest quarter | *Title and Bemmels,* [*2018*](http://envirem.github.io/) |
| **ENVIREM** | PETseasonality | monthly variability in potential evapotranspiration | *Title and Bemmels,* [*2018*](http://envirem.github.io/) |
| **ENVIREM** | PETWarmestQuarter | mean monthly PET of warmest quarter | *Title and Bemmels,* [*2018*](http://envirem.github.io/) |
| **ENVIREM** | PETWettestQuarter | mean monthly PET of wettest quarter | *Title and Bemmels,* [*2018*](http://envirem.github.io/) |
| **ENVIREM** | thermInd | compensated thermicity index: sum of mean annual temp., min. temp. of coldest month, max. temp. of the coldest month, x 10, with compensations for better comparability across the globe | *Title and Bemmels,* [*2018*](http://envirem.github.io/) |
| **ENVIREM** | tri | terrain roughness index | *Title and Bemmels,* [*2018*](http://envirem.github.io/) |
| **ENVIREM** | topoWet | SAGA-GIS topographic wetness index | *Title and Bemmels,* [*2018*](http://envirem.github.io/) |
| **gridded National Soil Survey Geographic (gNATSGO)** | awc_gNATSGO | Soil Available Water Capacity | *Boiko, Kagone, and Senay,* [*2021*](https://www.sciencebase.gov/catalog/item/5fd7c19cd34e30b9123cb51f) |
| **gNATSGO** | fc_gNATSGO | Soil Field Capacity | *Boiko, Kagone, and Senay,* [*2021*](https://www.sciencebase.gov/catalog/item/5fd7c19cd34e30b9123cb51f) |
| **gNATSGO** | por_gNATSGO | Soil Porosity | *Boiko, Kagone, and Senay,* [*2021*](https://www.sciencebase.gov/catalog/item/5fd7c19cd34e30b9123cb51f) |
| **daylength (geosphere)** | daylength_on_172 | Daylength of the longest day in the Northern Hemisphere (June 21) | *Hijmans,* [*2022*](https://cran.r-project.org/package=geosphere) |
| **daylength (geosphere)** | daylength_on356 | Daylength on the shortest day in the Northern Hemisphere (December 22) | *Hijmans,* [*2022*](https://cran.r-project.org/package=geosphere) |

Table 4.

| ENVIREM_PETseasonality | Monthly variability in potential evapotranspiration |
| --- | --- |
| ENVIREM_growingDegDays5 | Sum of mean monthly temperature for months with mean temperature greater than 5??C multiplied by number of days Sum of mean monthly temperature for months with mean temperature greater than 5??C multiplied by number of days Sum of mean monthly temperature for months with mean temperature greater than 5??C multiplied by number of days |
| ENVIREM_monthCountByTemp10 | Count of the number of months with mean temp greater than 10C |
| ENVIREM_topoWet | SAGA-GIS topographic wetness index |
| ENVIREM_tri | Terrain roughness index |
| awc_gNATSGO_US | Available Water Content |
| fc_gNATSGO_US | Field Capacity |
| por_gNATSGO_US | Porosity |
| wc2_1_2_5m_bio_8 | Mean temperature of wettest quarter |
| wc2_1_2_5m_elev | Elevation |
| wc2_1_2_5m_prec_01 | Precipitation in January |
| wc2_1_2_5m_prec_05 | Precipitation in May |
| wc2_1_2_5m_prec_06 | Precipitation in June |
| wc2_1_2_5m_prec_07 | Precipitation in July |
| wc2_1_2_5m_prec_09 | Precipitation in September |
| wc2_1_2_5m_srad_04 | Solar Radiation in April |
| wc2_1_2_5m_wind_12 | Wind Speeds in December |


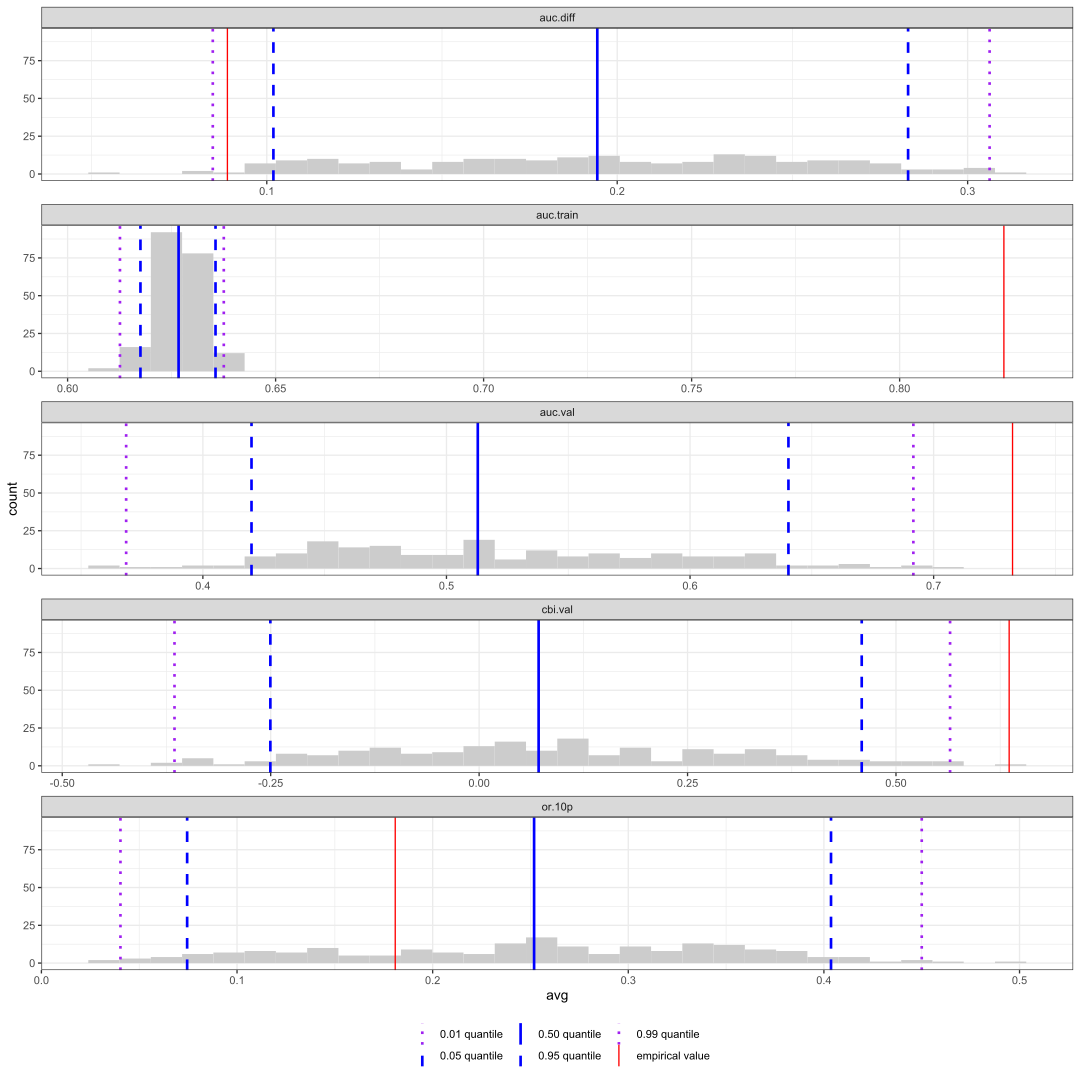

Figure 1. Graph of p-values generated from null models to identify significant validation statistics. Each separate graph represents one of five statistics. The empirical value of the p-values are represented by a solid red line. For positive statistics, AUC_test_, AUC_train_, and CBI, significance is to the right of the rightmost blue dashed line. For negative statistics, AUC_diff_ and OR_10p_, significance is to the left of the leftmost blue dashed line.


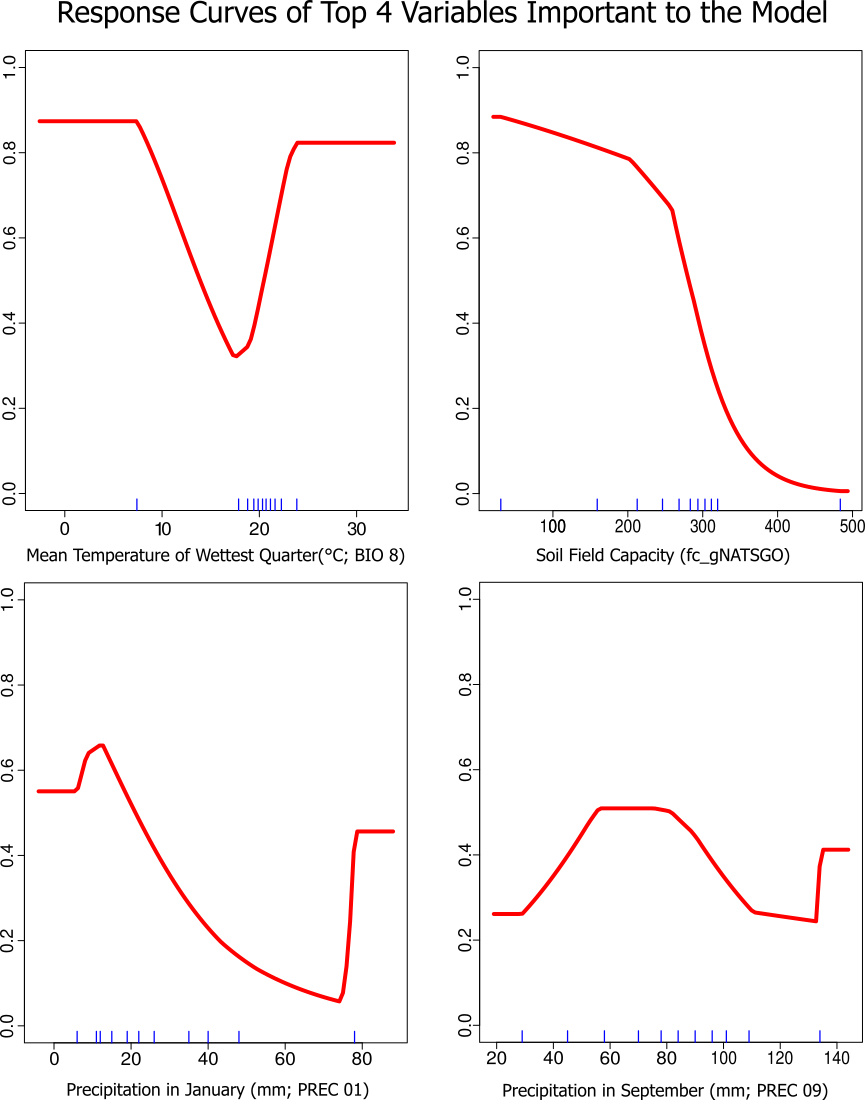


Figure 2. Environmental predictors response curves of the optimal model with individual predictor variable correlation with subpopulation suitability.

Figure 5.
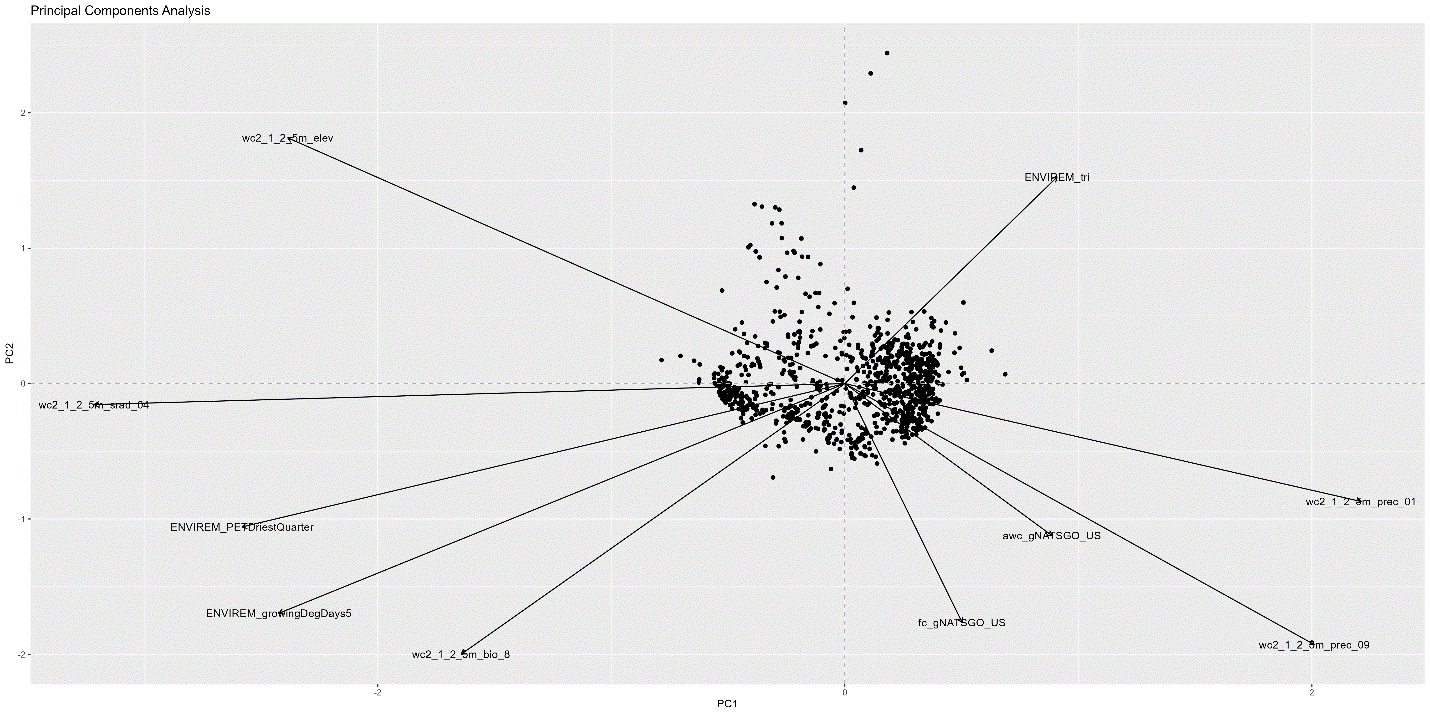


Reasoning 1.

Literature on an appropriate evaluation method to select optimal models is conflicting (Warren et al, [[2013](https://doi.org/10.1111/ddi.12160)](https://doi.org/10.1111/ddi.12160); Velasco and González-Salazar, [[2019](https://doi.org/10.1016/j.ecoinf.2019.02.005)](https://doi.org/10.1016/j.ecoinf.2019.02.005)). The first of two methods, the sequential method is cross validated by selectingmodels with the lowest 10% training omission rate and highest AUC average. The second method does not consider cross-validation, rather selects the model with the lowest ΔAIC_c_. Warren et al. ([2013](https://doi.org/10.1111/ddi.12160)) criticizes the use of AUC to evaluate Maxent models, as AUC overestimates model performance if testing and training data suffer similar sampling biases, concluding that AUC is an unreliable statistic to evaluate ENMs. Velasco and González-Salazar ([2019](https://doi.org/10.1016/j.ecoinf.2019.02.005)) disagree, stating that AIC should not be used as a selection criterion due to low predictive accuracy in the resulting models comparative to other statistics such as Kappa or TSS. However, they ([2019](https://doi.org/10.1016/j.ecoinf.2019.02.005)) also state that AICc selected models still need to be evaluated for their ability to select models that maximize explanatory power; the model that best describes the fundamental niche.

Using AICc score to select models typically identifies models that are simpler, with less parameters, and with less overfitting. However, the optimal model selected has a complex feature class and a low regularization multiplier, indicating the potential of overfitting. This must be validated through omission rates, in addition to other evaluation statistics to measure, classification capacity, discriminatory ability, and calibration. Null models generated indicate that the optimal model has good discriminatory ability but may be overfit.

AUC_test_ (or AUC_val_) scores averaged 0.53 across the 200 null models and typical null models score around 0.50 (Bohl, Kass, and Anderson ([2019](https://doi.org/10.1111/jbi.13573))). The optimal model was significant (p < 0.05) so the AUC_test_ is a significant statistic of model discrimination.
